# Changes in Viewer Engagement and Accessibility of Popular Vaping Videos on TikTok: A 12-Month Prospective Study

**DOI:** 10.3390/ijerph19031141

**Published:** 2022-01-20

**Authors:** Brienna N. Rutherford, Tianze Sun, Carmen C. W. Lim, Jack Chung, Brandon Cheng, Lily Davidson, Calvert Tisdale, Janni Leung, Daniel Stjepanović, Jason P. Connor, Gary C. K. Chan

**Affiliations:** 1 National Centre for Youth Substance Use Research, The University of Queensland, St. Lucia 4067, Australia; tianze.sun@uq.net.au (T.S.); c.lim@uq.net.au (C.C.W.L.); yiuchak.chung@uq.edu.au (J.C.); brandon.cheng@uq.edu.au (B.C.); j.leung1@uq.edu.au (J.L.); d.stjepanovic@uq.edu.au (D.S.); jason.connor@uq.edu.au (J.P.C.); c.chan4@uq.edu.au (G.C.K.C.); 2 School of Psychology, The University of Queensland, St. Lucia 4067, Australia; lily.davidson@uq.net.au (L.D.); c.tisdale@uq.edu.au (C.T.); 3 National Drug and Alcohol Research Centre, University of New South Wales, Kensington 2033, Australia; 4 Discipline of Psychiatry, The University of Queensland, Herston 4006, Australia

**Keywords:** e-cigarettes, vaping, TikTok, social media

## Abstract

Aim: There are concerns regarding what young people are exposed to on TikTok due to trending content promoting e-cigarette use through humour, marketing and lifestyle acceptability. Using baseline data from November 2020, we aimed to examine how much content from a sample of popular vaping videos remained accessible at 9- and 12-month follow-ups. We aimed to monitor changes in viewer engagement (using metadata) before and after the U.S. Congressional Hearing on youth protection measures on social media in October 2021. Methods: Hashtag-based keywords were used to collect the most viewed publicly available e-cigarette related videos on TikTok (N = 802) from inception to November 2020 to form a baseline. Researchers conducted a longitudinal descriptive study using this data, with 9- and 12-month follow-ups to measure changes in viewer engagement (using metadata) and content availability. Findings: Of the 802 videos from the baseline, 562 remained at the 9-month follow-up and 511 remained at the 12-month follow-up. At the 12-month follow-up, the majority of vaping-related hashtags were removed by TikTok after the Congressional Hearing. Between the baseline and 9-month follow up, views increased by 1.4% and likes increased by 4.4%. At 12-month follow-up, views had increased by 1.7% and likes by 4.2% compared to baseline data. Whilst 291 videos were no longer publicly accessible at 12-month follow-up, 39 of these were made inaccessible by the content creators. The most viewed and most liked vaping videos at baseline were still publicly available. Conclusions: Whilst the depiction type and thematic distribution of removed videos suggest that TikTok may be removing a small proportion of content that promotes the use of e-cigarettes, metadata of remaining videos indicate an increase in viewer engagement. TikTok’s removal of explicit substance-related hashtags from the platform could be a step towards preventing adolescents from being exposed to harmful behaviours and substances online. However, the platform should consider enforcing effective age restrictions on content that promotes substance use in a positive light.

## 1. Introduction

Adolescent electronic-cigarette (e-cigarette) use is increasing, with self-reported use exceeding that of traditional cigarettes [1] in many developed countries. In 2021, current e-cigarette use (past 30-day use) was reported by 7.6% of all middle and high school students [2]. One possible reason for this increase in use is the availability of flavoured and discretely designed e-cigarette products. Flavour varieties, such as fruit or candy, are appealing to adolescent users and are often the primary reason for initial experimentation with vaping [3]. Additionally, adolescent e-cigarette non-users and users are more likely to perceive vaping as a safer alternative to traditional cigarette use and underestimate the potential risks associated with regular use [4]. An estimated 66% of JUUL (a specific brand of e-cigarette) users between the ages of 15 to 24 years were unaware that JUUL products contain nicotine [3].

With e-cigarettes widely promoted on the internet [5] through advertisements [6], social media influencers [7] and positive user-generated sentiments [8], it is likely that adolescents who are active social media users may be at higher risk for subsequent e-cigarette use through perceived normalisation and social acceptability [9]. Increased adolescent social media use has also been associated with decreased perceived risk of use and desensitisation to associated harms [9]. Recent findings have also indicated that youth consider online para-social interactions on social media to be highly similar to their in-person social interactions and relationships [10]. Therefore, young social media users are more likely to have their attitudes and behaviours influenced by peers or influencers on social media.

Recent content analyses of TikTok [11,12], Instagram [13] and YouTube [14,15,16] have identified a high prevalence of pro-e-cigarette use and vaping-related content. This is despite community guidelines on many social media platforms prohibiting the depiction, promotion or trade of drugs or other controlled substances, such as alcohol or tobacco products [17]. In 2020, a content analysis found that the top 10 TikTok videos on Puff Bar e-cigarettes (42.4 million views) were mostly related to inhaling nicotine or nicotine addiction, with two videos promoting the sale of e-cigarettes, four videos trivialising nicotine addiction, one Puff Bar repair tutorial and another two explicitly portraying underage youth using e-cigarettes [12]. These videos were viewed between 2.8 and 42.4 million times [12]. Exposure to this content could lead to increased intentions to initiate use and increased risk of normalization [18].

Content regulation algorithms are key to social media platforms being able to maintain their community guidelines and the online safety of their users by removing content that breaches their terms and conditions [19]. However, content regulation is complicated by the need for platforms to maximise user time on the platform through “explosive” or polarising content [20]. Although exposure to pro-vaping related content is likely to increase the likelihood for future vape use, many online platforms, such as TikTok, are increasingly relying on an “attention economy”—a marketing perspective valuing something’s capacity to attract attention [20]. The effect that exposure to substance use imagery on social media platforms may have on the behaviour and attitudes of adolescents has become the focus of a recent (October 2021) United States (U.S.) Congressional Hearing [21] in the Senate. The U.S. Senate’s Commerce Committee launched a consumer protection panel to investigate how social media platforms such as YouTube, Snapchat and TikTok may affect children and what these companies are doing to limit exposure to harmful behaviours, bullying and substances online [21]. At these hearings, these social media companies reported on their artificial-intelligence algorithms and claimed to commit to additional moderators to remove potentially harmful content [22]. For example, TikTok has announced that all content will be screened using its automated policy violation detection tools as part of the upload process in addition to existing content self-regulation processes employed by TikTok, which include their flagging system (where content is flagged by an automated process and reviewed by an internal U.S.-based safety team) [23]. TikTok has also removed substance-related hashtags and limited searches for keywords explicitly related to drugs or other substances (such as ‘vape’, and ‘cannabis’). However, substance use content continues to proliferate on the platform and remains publicly accessible [22] by using modified spelling wherein numbers replace letters (e.g., ‘w33d’ and ‘vap3’) to avoid detection from text-based regulation algorithms. 

With more than one billion active monthly users [21], TikTok is one of the most popular social media platforms among young people and has continued to surge in popularity due to its convenient-to-use platform and short-form video content [24]. This popularity is further enhanced by a recommendation algorithm that gives the impression that the content has been personally tailored for the user [25,26]. Due to both its continuing popularity among adolescents and young adults, TikTok was chosen as the platform of focus for the current study. This study aims to follow-up a previous content analysis study [11] to monitor changes in availability of vaping-related videos, viewer engagement (using publicly available metadata), themes and sentiment of remaining and removed vaping videos on TikTok at 9- and 12-month follow-ups. The U.S. Congressional Hearing on social media platforms was held in October 2021, which was 12 months after our baseline data collection. This study will examine changes in vaping content on TikTok before and after the hearing. 

## 2. Method

### 2.1. Sampling Strategy

At baseline in November 2020, hashtag-based keywords on publicly available vaping related videos (N = 802) were used to collect content from TikTok. When authors revisited the URLs used in the baseline study, six videos were identified as duplicates due to having been extracted using mobile-applications rather than Internet browsers. Agreement to include videos was calculated on a subset of 100 videos for the original paper. Specific information on the hashtag-based keywords and their corresponding views can be found in Table S1 in the Supplementary Materials.

This content was then thematically analysed to determine how vaping-related videos were being depicted to viewers and assessed for sentiment and age-restriction warnings. Publicly available URL links for each video, metadata (i.e., likes and views) and perceived presenter demographics were also collected. Using this baseline sample, 9- and 12-month follow-ups were conducted using the URL links to access content (see [11] for details).

### 2.2. Coding Procedures

Baseline data were collected in November 2020 (N = 802) and coded for sentiment and thematic information. Sentiment was coded as positive, negative, or neutral and seven non-mutually exclusive themes were identified: ‘comedy and joke’, ‘marketing’, ‘lifestyle and acceptability’, ‘vaping tricks’, ‘nicotine and addiction’, ‘creativity’ and ‘warning’. Sentiment and themes of videos (remaining and removed) at 9- and 12-month follow-ups were reported. 

**Figure 1:**
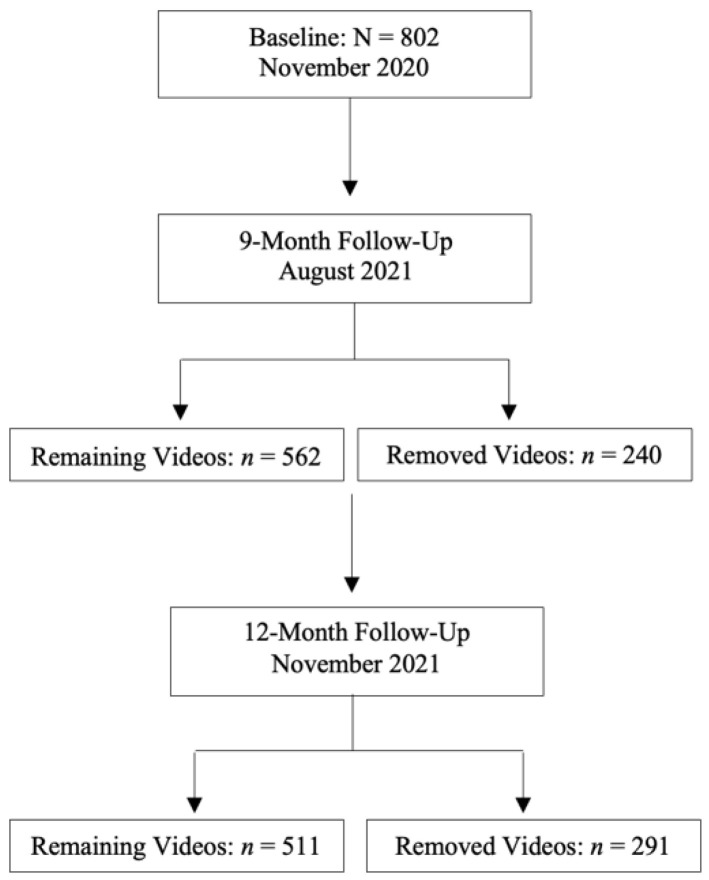
Sample Sizes.

One of the authors (BR) followed the URL links from the baseline study (N = 802) to access these videos in August 2021 (9-month follow-up), to determine whether the content was still publicly available. Where videos had been removed by TikTok, the URL would display a message to the viewer that read “Video currently unavailable” rather than the actual video content. Videos that were initially uploaded as publicly accessible at baseline but had since been privatised by the user displayed a message that read “This video is private”. Updated metadata (likes and views) were also collected to determine if there was an increase in viewer engagement since the original data collection period. The usernames of content creators were also searched using TikTok’s search function to determine if the accounts were still active on the platform or had been reactivated with slight alterations to their username (e.g., addition of a letter or numeric digit). This process was repeated in November 2021 (12-month follow-up). In total, 562 and 511 videos remained at 9- and 12-month follow-ups, respectively. Baseline data pertaining to metadata, sentiment and themes were also used to report results of removed videos at 9- (*n* = 240) and 12-month (*n* = 291) follow-ups (refer to Figure 1).

We calculated the change in views and likes between baseline and 9- and 12-month follow-ups by comparing the mean likes and engagements for the videos still publicly available (*n =* 511) at 12-month follow-up. This was to ensure we were capturing actual changes in viewer engagement and not just changes attributable to the removal of videos in our sample. 

Ethics exemption was obtained from the Office of Research Ethics at The University of Queensland (exemption ref: 20200011080). Videos were streamed only through publicly available URLs, and no media content was locally downloaded, copied or modified. 

## 3. Results

### 3.1. Changes Reflected at 9-Month Follow-Up (before October 2021 Congressional Hearing)

**Remaining Videos at 9-Month Follow-Up.** Of the 802 videos in the original sample, 562 (70.1%) remained publicly available in August 2021. These videos had a mean view count of 2,061,940 (SD *=* 2,877,891) and a mean ‘likes’ count of 297,504 (SD = 549,551). No videos in the baseline sample (N = 802) displayed age restriction warnings, and we observed no age restriction warnings in the remaining sample at 9-month follow-up. Between the baseline and 9-month follow-up (using the final 12-month sample, *n* = 511), likes increased from 286,766 to 299,397 (1.4%) and views increased from 2,022,787 to 2,050,275 (4.4%).

Among the remaining videos (*n* = 562), content using humour to promote vaping was most prominent (48.8%), followed by content promoting the lifestyle acceptability of vaping (29.4%), marketing content (20.1%), vaping tricks (18.7%), videos mentioning nicotine or addiction (18.7%), informative or creative tutorials (14.6%) and lastly, content warning users about associated health risks (11.4%). Videos predominantly depicted vaping positively (52.7%). An additional 22.9% depicted vaping neutrally and 14.2% depicted vaping negatively. Refer to Table S2 in the Supplementary Materials for further information. 

**Removed Videos at 9-Month Follow-Up.** Whilst 240 (29.9%) videos were no longer publicly available, 33 (13.8%) of these had since been privatised by the content creator and were not removed by TikTok’s content moderation. These removed videos had a mean view count of 1,771,496 (SD = 2,922,273) and a mean ‘likes’ count of 263,155 (SD = 548,481) when initially collected from the platform in November 2020. Among the removed videos, 109 (45.4%) of the removed videos had more views than the total sample median views (*n* = 1,000,000), and 119 (49.6%) of the removed videos had more likes than the total sample median likes (*n* = 143250). 

We found that 74.6% of the removed videos depicted vaping positively. A further 18.7% of videos depicted vaping neutrally and a final 7.5% of videos depicted vaping negatively. Active vaping use was depicted in 60 (25%) of the removed videos, and 190 (79.2%) removed videos showed one or more vaping devices. Among the remaining videos (*n* = 562), content using humour to promote vaping was most prominent (46.3%), followed by content promoting the lifestyle acceptability of vaping (42.1%), marketing content (38.3%), vaping tricks (21.3%), videos mentioning nicotine or addiction (17.1%), informative or creative tutorials (13.3%) and lastly, content warning users about associated health risks (6.7%). Refer to Table S3 in the Supplementary Materials for further information.

### 3.2. Changes Reflected at 12-Month Follow-Up (after October 2021 Congressional Hearing) 

**Remaining Videos at 12-Month Follow-Up.** Following the U.S. Congressional Hearing in October 2021, 511 (63.71%) of the original videos remained publicly accessible despite TikTok’s post-Congressional Hearing actions which removed hashtags that explicitly referenced vaping behaviours. Removal of hashtags results in users not being able to search terms, such as ‘vaping’ or ‘vape tricks’ to find collections of videos and receiving a warning that these terms may breach community guidelines on the platform. Of the nine hashtag-based keywords used to identify vaping-related videos at baseline (see Table S1 in Supplementary Materials for a complete list of hashtags), only #juulgang remained publicly accessible. However, videos stored under these removed hashtags were not necessarily removed. Again, no age restriction warnings had been added to the content. These remaining videos had a mean view count of 2,059,617 (SD *=* 2,886,899) and a mean ‘likes’ count of 299,356 (SD = 565,157). At 12-month follow-up (using the final 12-month sample, *n* = 511), views had increased from 2,022,787 to 2,057,052 (1.7%) and likes from 286,766 to 298,925 (4.2%). Between 9- and 12-month follow-ups, views increased from 2,050,275 to 2,057,052 (0.3%) and likes decreased from 299,397 to 298,925 (3.7%). Additionally, the most viewed and most liked videos were still publicly available. 

Videos depicting vaping in a humorous way were most common in the remaining publicly available videos (*n* = 511) and accounted for 54.6% of the sample at 12-month follow-up. Content promoting the lifestyle acceptability of vaping use was the next most prominent (33.1%), followed by marketing material (22.5%), videos mentioning nicotine addiction (20.7%, *n* = 106), vaping trick tutorials (20.5%) and creativity tips (16.2%). Videos warning users about the risks of vaping were the least common (12.5%). A total of 122 (23.9%) videos depicted individuals actively vaping, with 59.7% videos showing one or more vaping products in the video. Videos predominantly depicted vaping positively (58.5%), with only 16.0% of videos depicting vaping negatively and a further 25.4% portraying vaping neutrally. Refer to Table S2 in the Supplementary Materials for further information.

**Removed Videos at 12-Month Follow-Up.** A total of 291 (36.3%) videos were no longer publicly accessible, with 39 (13.4%) of these privatised by content creators and not by TikTok’s content regulation algorithms. These removed videos had a mean view count of 1,745,926 (SD = 2,633,559) and a mean ‘likes’ count of 252,306 (SD = 504,006) when initially collected from the platform in November 2020. Among the removed videos, 131 (45.0%) of removed videos exceeded the total sample median views (*n* = 1,000,000) and 144 (49.5%) of the removed videos exceeded the total sample median likes (*n* = 143,250). 

We found that 73.2% of the removed videos (*n* = 291) depicted vaping positively. A further 18.9% videos depicted vaping neutrally and a final 7.9% videos depicted vaping negatively. Content using humour to promote vaping was most prominent (48.8%), followed by marketing (39.5%) and videos promoting the lifestyle acceptability of vaping (39.2%). Videos featuring vaping tricks (18.9%), references to nicotine addiction (18.2%), creativity tips (16.2%) and warning of associated risks of use (7.7%) were less common. Comparisons of depiction types and theme distributions of removed videos at 9- and 12-month follow-ups can be found in Table S3 in the Supplementary Materials. 

It is also interesting to note that seven videos that had been previously removed by TikTok had become publicly available again at the 12-month follow-up. This is likely due to the content having presumably gone through content moderation and being deemed appropriate for viewing.

## 4. Discussion

This study measured changes in availability, viewer engagement (using metadata), themes and sentiment of popular vaping videos on TikTok. Our analysis revealed that the majority of e-cigarette and vaping-related content identified in a previous TikTok content analysis [11] was still publicly available at 9- and 12-month follow-ups. Videos did not display age restriction warnings to prevent adolescent and young adult exposure despite TikTok’s additional self-regulated algorithms, moderators and removal of hashtag-keywords. 

Whilst most of the removed videos were pro-vaping, indicating that TikTok’s content moderation is removing content that may have consequences on adolescent attitudes and subsequent use, a large portion of pro-vaping and e-cigarette content was still publicly accessible. Existing studies have found that higher frequency of social media use is associated with higher rates of vaping among young adults [27] and adolescents [28]. Availability of unrestricted content promoting the use of e-cigarettes to a young audience not only has the capacity to influence substance use attitudes and behaviours [9] but also opposes current regulatory approaches towards e-cigarettes [29]. In Australia, the manufacture, sale or supply of e-cigarettes containing nicotine without authority (e.g., medical prescription) is prohibited due to the classification of liquid nicotine as a Schedule 7–Dangerous Poison [30]. Vaping-related content on the TikTok platform has been found to encourage use and promote vaping as a socially acceptable alternative to traditional smoking [11], which minimises the associated health risks to viewers. 

Additionally, of the accounts removed, e-cigarette and vaping-specific accounts had resurfaced under subtly different usernames. This indicates that TikTok’s moderation does not sufficiently identify and restrict duplicate accounts (which would likely be using the same account details) that promote vaping or e-cigarette use. This means that the content promoting vaping and e-cigarette use will continue to resurface despite self-regulation attempts. This could serve to minimise perceptions of associated health risks and allow promotional e-cigarette and vaping-related content to put forth a positive image unfettered by contemporary health research [29]. 

Less than half of the removed videos were in the upper percentile of views and likes, indicating the content regulation algorithm on TikTok does not specifically target content with high engagement. Content with high engagement (e.g., higher likes and views) is likely to have further reach among platform users [31]. Therefore, content regulation algorithms that do not target high engagement videos result in a higher risk of adolescent and young adult exposure to positive e-cigarette and vaping-related content by allowing these videos to remain publicly accessible on the platform. Additionally, whilst TikTok has removed hashtag-keywords such as #vape and its derivatives (e.g., #vaping, #vapetricks), the most viewed vaping-related hashtag (#juulgang) remains active, and all content previously stored under the removed hashtags is also still publicly available under alternate hashtags. Whilst it may seem a logical first step to remove hashtags that explicitly reference substances by name, this does little to reduce the availability of substance-related content on TikTok. 

Social media sites, such as TikTok, have come under increased scrutiny in recent years for their lack of action to protect adolescent users from exposure to harmful or risky content [22]. Mandatory age restrictions on content related to the use of substances is likely to be more effective than restricting direct messaging ability, as is currently done on TikTok [21]. Additionally, platforms should focus on removing highly viewed hashtags as well as keyword-hashtags to limit the reach of potentially harmful content. TikTok’s new content regulation algorithm is a move in the right direction in terms of restricting content that promotes substance use; however, further refinements are clearly required, and follow-up studies should be undertaken to evaluate its long-term efficacy. 

A limitation of the present work is that our study can only determine whether removed videos and accounts were taken down voluntarily by content creators or by TikTok’s content regulation for a subset of the sample. Videos or accounts that were privatised and accounts that generated a breached community guidelines warning can be confidently determined to be removed by the user or TikTok, respectively. However, we can only hypothesise based on the thematic makeup of the remaining videos that they were removed by TikTok for pro-vaping and e-cigarette use attitudes. Additionally, whilst we were able to identify accounts that had been reuploaded under subtly different usernames, we cannot identify whether removed videos were reuploaded under different hashtags or accounts. Therefore, the content may still be publicly available on the platform. Additionally, our comparisons between content availability and viewer engagement at 9- and 12-month follow-ups are confounded by time. The decline in availability could be more attributable to natural attrition on the same trajectory observed between the baseline and 9-months, rather than the actions resulting from the Congressional Hearing between 9 and 12-month follow-ups. Lastly, the videos identified as ‘most viewed’ vaping videos may no longer have been the most viewed videos at follow-up. Continuous monitoring is needed to ascertain the availability and accessibility of newly created vaping videos. 

## 5. Conclusions

From our sample of vaping-related videos, more than half were still publicly accessible, which suggests that although TikTok’s current content regulation model is an improvement, this system is ineffective for identifying most content depicting substance use. Given that previous research demonstrated that exposure to vaping-related content may have consequences on attitudes and subsequent use behaviours among adolescents and young adults, it is imperative that better content regulations are implemented. At the very least, warnings against engaging in such behaviours should be displayed on substance use content. 

## Data Availability

Data available on request due to privacy restrictions.

## Supplementary Materials

The following are available online at https://www.mdpi.com/article/10.3390/ijerph19031141/s1, Table S1: Hashtag-Based Keywords and Corresponding Views (in Millions) at Baseline, Table S2: Comparisons of Metadata, Sentiment and Themes in Remaining Available Videos at the Baseline and 9- and 12-Months, Table S3: Comparisons of Metadata, Sentiment and Themes in Removed Videos at the Baseline and 9- and 12-Months.
