# Supplementary material for: Changes in Viewer Engagement and Accessibility of Popular Vaping Videos on TikTok: A 12-Month Prospective Study
Source: Int J Environ Res Public Health. 2022 Jan 20;19(3):1141. doi: 10.3390/ijerph19031141 (PMC8834819; doi:10.3390/ijerph19031141)
Supplement: Supplementary file 1 [file ijerph-19-01141-s001.zip › ijerph-1543739-supplementary.pdf]

International Journal of
Environmental Research and Public Health

MDPI

Supplementary Material

Supplementary Materials

Table S1. Hashtag-Based Keywords and Corresponding Views (in Millions) at Baseline.

Hashtag-Based Keywords	Views (in Millions) in November 2020
#juulgang	587.7
#nicotine	312.5
#puffbar	348.2
#vape	2300
#vapelife	242.6
#vapenation	328.9
#vapeshop	283.2
#vapetricks	868.6
#vaping	279.6

Table S2. Comparisons of Metadata, Sentiment and Themes in Remaining Available Videos at the Baseline and 9- and 12-Months.

Variable	Baseline Sample (n = 802)	9-Month Sample (n = 562)	12-Month Sample (n = 511)
**Metrics**			
Total Media Views	2022787 (SD = 4300294)	2061940 (SD = 2877891)	2059617 (SD = 2886899)
Total Media Likes	286766 (SD = 566620)	297504 (SD = 549551)	299356 (SD = 565157)
**Sentiment**			
Positive	510 (63.6%)	296 (52.7%)	299 (58.5%)
Negative	188 (23.4%)	80 (14.2%)	82 (16.0%)
Neutral	104 (13.0%)	129 (22.9%)	130 (25.4%)
**Themes**			
Comedy and Joke	420 (52.4%)	274 (48.8%)	279 (54.6%)
Lifestyle Acceptability	270 (34.8%)	165 (29.4%)	169 (33.1%)
Marketing	229 (28.6%)	113 (20.1%)	115 (22.5%)
Vaping Tricks	159 (19.8%)	105 (18.7%)	105 (20.5%)
Nicotine and Addiction	159 (19.8%)	105 (18.7%)	106 (20.7%)
Creativity and Tips	129 (16.1%)	82 (14.6%)	83 (16.2%)
Warning	85 (10.6%)	64 (11.4%)	64 (12.5%)

Table S3. Comparisons of Metadata, Sentiment and Themes in Removed Videos at the Baseline and 9- and 12-Months.

Variable	Baseline Sample (*n* = 802)	9-Month Sample (*n* = 240)	12-Month Sample (*n* = 291)
**Metrics**			
Total Media Views	2022787 (SD = 4300294)	1771496 (SD = 2922273)	1745926 (SD = 2633559)
Total Media Likes	286766 (SD = 566620)	263155 (SD = 548481)	252306 (SD = 504006)
**Sentiment**			
Positive	510 (63.6%)	179 (74.6%)	213 (73.2%)
Negative	188 (23.4%)	18 (7.5%)	23 (7.9%)
Neutral	104 (13.0%)	45 (18.7%)	55 (18.9%)
**Themes**			
Comedy and Joke	420 (52.4%)	111 (46.3%)	142 (48.8%)
Lifestyle Acceptability	270 (34.8%)	101 (42.1%)	114 (39.2%)
Marketing	229 (28.6%)	92 (38.3%)	115 (39.5%)
Vaping Tricks	159 (19.8%)	51 (21.3%)	55 (18.9%)
Nicotine and Addiction	159 (19.8%)	41 (17.1%)	53 (18.2%)
Creativity and Tips	129 (16.1%)	32 (13.3%)	47 (16.2%)
Warning	85 (10.6%)	16 (6.7%)	22 (7.7%)
